# Heme Oxygenase 1‐Targeted Hybrid Nanoparticle for Chemo‐ and Immuno‐Combination Therapy in Acute Myelogenous Leukemia

**DOI:** 10.1002/advs.202000487

**Published:** 2020-06-03

**Authors:** Seok‐Beom Yong, Jaehyun Kim, Jee Young Chung, Sehee Ra, Seong Su kim, Yong‐Hee Kim

**Affiliations:** ^1^ Department of Bioengineering Institute for Bioengineering and Biopharmaceutical Research BK 21 Plus Future Biopharmaceutical Human Resources Training and Research Team Hanyang University Seoul 133‐791 Republic of Korea

**Keywords:** acute myelogenous leukemia, cancer immunotherapy, heme oxygenase 1‐targeted leukemia immunotherapy, heme oxygenase 1‐targeted nanomedicine, immunotherapeutic nanomedicine

## Abstract

Acute myelogenous leukemia (AML) is a fatal blood cancer with high patient mortality. Daunorubicin and cytarabine are first‐line chemotherapy for AML, with bone marrow transplantation in most cases. Recently, cancer immunotherapy has been challenged in AML and leukemia‐niche myeloid cells are promising targets for the AML immunotherapy. Heme oxygenase 1 (HO1) is an antioxidative and cytoprotective enzyme inducing chemo‐resistant AML and has been focused as an immune checkpoint molecule in tumor microenvironments. Herein, lipid‐polymer hybrid nanoparticle (hNP) is loaded with tin mesoporphyrin (SnMP), a HO1‐inhibitor, and non‐covalently modified with an engineered antibody for leukemic cell‐targeted delivery. HO1‐inhibiting T‐hNP (T‐hNP/SnMP) enhances chemo‐sensitivity in human leukemia cells. In a human AML‐bearing orthotopic mouse model, intravenously injected T‐hNP not only actively targets to human leukemia cells but passively targets to CD11b+ myeloid cells in a bone marrow niche. The T‐hNP/SnMP enhances the chemo‐therapeutic effect of daunorubicin and boosts immune response by reprogramming bone marrow myeloid cells resulting from the recruitment of the monocyte‐lineage and induction of inflammatory genes. The ex vivo study demonstrates an enhanced immune response of HO1‐inhibited bone marrow CD11b+ myeloid cells against apoptotic leukemia cells. Collectively, HO1‐inhibiting dual cell‐targeted T‐hNP/SnMP has a strong potential as a novel therapeutic in AML.

## Graphical abstract

The HO1‐targeted hybrid nanoparticle (T‐hNP/SnMP) is developed for chemo‐ and immuno‐combination therapy of acute myelogenous leukemia (AML). T‐hNP/SnMP enhances the chemo‐therapeutic effect of daunorubicin and boosts immune response by reprogramming bone marrow myeloid cells. This finding suggests that HO1‐inhibitng dual cell‐targeted hNP has a strong potential as a novel therapeutic in AML.

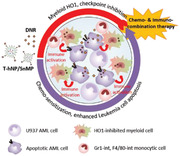

## 1 Introduction

**Scheme 1:**
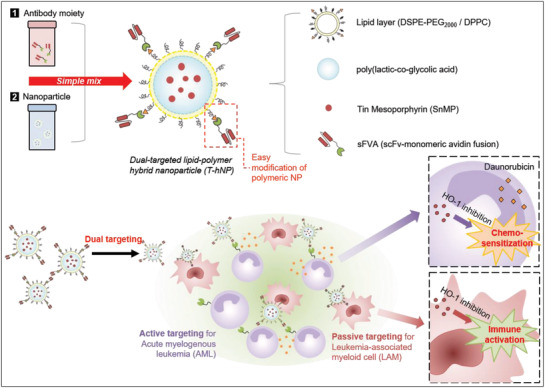
Schematic illustration of HO1‐inhibiting hybrid nanoparticle for chemo‐and immuno‐combination therapy in acute myeloid leukemia (AML). The hNP is actively targeted to AML cells by sFVA‐modification and passively targeted to LAM cells by negatively charged surface and phagocytic uptake.

Acute myelogenous leukemia (AML) is one of the most life‐threatening hematological malignancies. Approximately 10 000 patients died of AML in the US alone in 2018. Various genetic drivers and their combinations are involved in AML, and multiple leukemic clones that directly affect therapeutic responsiveness, relapse rates, and chemoresistance have been reported.^[^ ^1^ ^]^ Combinations of anthracycline drugs and cytarabine have been used for the treatment of AML and, followed by bone marrow transplant in most cases.^[^ ^2^ ^]^ Cancer immunotherapy based on immunological understanding of cancer‐immune cell interactions has been developed and clinically used, most successfully with PD‐1‐ and CTLA4‐targeted T cell‐activating strategies.^[^ ^3^ ^]^ However, most of cancer immunotherapies are limited to solid tumor models such as melanoma and breast tumor.^[^ ^3a^ ^]^ Recently, the effectiveness of anti‐PD‐1 therapy was demonstrated in relapsed AML patients,^[^ ^4^ ^]^ and other blood cancer models.^[^ ^5^ ^]^ In a tumor microenvironment, myeloid lineages such as monocytes and macrophages (TAMs) constitute supportive cells that compromise anti‐tumor immune responses by facilitating pro‐tumoral microenvironment development,^[^ ^6^ ^]^ and metastasis for secondary tumor evasion.^[^ ^7^ ^]^ TAMs are common in various types of tumors,^[^ ^7^ ^]^ and TAM‐targeted immunotherapy has been challenged using CSF1R inhibitors,^[^ ^8^ ^]^ and nanoparticle‐mediated TAM‐reprogramming in melanomas, colon cancers, breast tumors, and other solid tumors.^[^ ^9^ ^]^ In the case of blood cancer, leukemia‐education and attraction of TAMs have been identified in spleen and bone marrow niches of AML,^[^ ^10^ ^]^ and repolarization of bone marrow niche macrophages to an M1‐like phenotype has been reported to suppress growth of myeloma cells.^[^ ^11^ ^]^ More recently, CSF1R‐expressing, leukemia‐supportive myeloid lineages from AML patient samples has been proved indispensable for genetic heterogeneity,^[^ ^12^ ^]^ suggesting immunotherapeutic potential of myeloid cell (TAMs) reprogramming in AML. Heme oxygenase 1 (HO1) is an antioxidant, heme‐degrading enzyme representing protective effects in various tissues and HO1‐mediated chemo‐resistant mechanisms have been studied in various cancers,^[^ ^13^ ^]^ and AML samples from patients.^[^ ^14^ ^]^ In addition, HO1 siRNA‐mediated chemo‐sensitization has been validated in a human AML orthotopic model.^[^ ^15^ ^]^ On the other hand, HO1‐knockout macrophages represented more M1‐like and inflammatory gene expressions,^[^ ^16^ ^]^ and a recent study by Muliaditan et al.^[^ ^17^ ^]^ reported tumor myeloid cell‐specific HO1 expression which was pharmacologically controlled with HO1 inhibitors, inducing an anti‐tumor immune response in breast tumor model, suggesting HO1 can act as a novel immune checkpoint molecule. However, the immunotherapeutic effects of HO1 inhibition have not been validated in blood cancer models, such as AML. Due to the ease of modification, drug loading, and tumor‐targeting effect, nanoparticle‐based cancer immunotherapies were proven to be effective.^[^ ^18^ ^]^ In this study, lipid‐polymer hybrid nanoparticles (hNP) were loaded with Tin mesoporphyrin (SnMP), a HO1‐inhibitor, and non‐covalently modified with an engineered single chain antibody (T‐hNP) for active targeting of acute myeloid leukemia cells and passive targeting to leukemia‐associated myeloid cell (LAM), and T‐hNP‐mediated synergistic dual therapeutic effects combining chemo‐sensitization of AML and immuno‐reprogramming of LAM were evaluated in orthotopic xenograft model (**Scheme** **1**).

## 2 Results

### 2.1 Preparation and Characterization of Engineered Antibody Fusion Protein, sFVA

**Figure 1:**
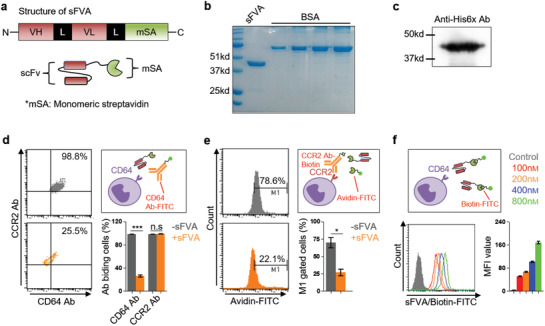
Preparation and characterization of engineered antibody fusion protein, sFVA. a) Structure of the antibody fusion protein sFVA. An scFv‐encoding sequence was inserted between XbaI and NotI followed by GS‐linker and monomeric streptavidin (msA). b) SDS‐PAGE data of purified sFVA fusion protein. c) His‐tag immunodetection of sFVA. The sFVA represents a molecular weights of ≈43 kDa. d) A competition assay with anti‐CD64 monoclonal antibody. THP‐1 cells were analyzed for anti‐CD64 and ‐CCR2 mAb binding after pre‐treatment of sFVA (10 µg mL^−1^). e) A competition assay with avidin‐FITC for biotin binding. The anti‐CCR2 mAb‐biotin was or was not bound with avidin‐FITC in the presence of sFVA and analyzed for cell binding. f) Concentration‐dependent cell binding of sFVA/biotin‐FITC. The biotin‐FITC‐bound sFVA was analyzed for THP‐1 cell binding. Data are presented as mean ± SD. All statistical analyses were performed with a Student's *t*‐test, *p < 0.05 ***p < 0.001, *n* = 3–6 per group.

Recently, non‐covalent antibody modification of nanoparticles has been demonstrated as an efficient targeted delivery strategy,^[^ ^19^ ^]^ and biotin‐avidin affinity is particularly selective.^[^ ^20^ ^]^ Since anti‐human CD64‐targeting single chain antibody (scFv) was found to be effective in human leukemia cell‐targeted delivery in vivo,^[^ ^15^ ^]^ anti‐CD64 scFv was recombinantly fused to monomeric avidin^[^ ^21^ ^]^ (sFVA) for nanoparticle modification (**Figure** **1**a). The sFVA was expressed and purified from a bacterial expression system and dialyzed to recover its antigen‐binding ability. As shown in Figure 1b,c, the sFVA band was identified at the molecular weight of ≈43 kDa, which is consistent with its theoretically estimated molecular weight. To evaluate human CD64‐ and biotin‐binding abilities, sFVA was competed with commercial antibodies. The CD64‐expressing THP‐1 cells showed reduced anti‐CD64 antibody‐FITC binding in the presence of sFVA with 3.75‐fold lower % of cell binding compared to the non‐competed group, while anti‐CCR2 antibody‐PE did not compete with sFVA (Figure 1d). A competition assay with anti‐CCR2 mAb‐biotin and avidin‐FITC proved the biotin‐binding affinity of sFVA with 2.6‐fold lower % of cell binding in the presence of sFVA compared to the non‐competed group (Figure 1e). Biotin‐FITC‐bound sFVA exhibited concentration‐dependent cell binding profiles (Figure 1f).

### 2.2 Optimization and Characterization of PLGA‐Lipid Hybrid Nanoparticles

**Figure 2:**
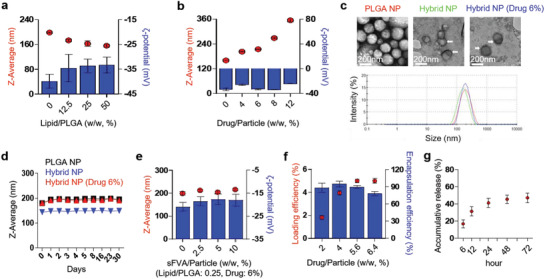
Optimization and characterization of PLGA‐lipid hybrid nanoparticle (hNP). a) Optimization of the lipid to PLGA ratio for hybrid nanoparticle preparation. The hybrid nanoparticle was synthesized in various amounts of lipid to PLGA ratios (DPPC:DSPE‐PEG_2000_, 3:1). b) Optimization of HO1 inhibitor to PLGA ratio for hybrid nanoparticle preparation. c) Scanning electron microscopy image of empty‐ and SnMP‐loaded‐ hybrid nanoparticles. The hybrid nanoparticles were lyophilized and imaged. d) Stability test of the hybrid nanoparticle. The prepared and concentrated hybrid nanoparticles were analyzed by a Zeta‐Sizer at the indicated days. e) Optimization of sFVA‐bound hybrid nanoparticles. f) Drug loading efficiency and encapsulation efficiency test. g) Drug release test. All data are presented as mean ± SD, *n* = 3–6 per group.

Lipid‐layered polymeric hNPs have been reported as efficient drug delivery carriers for cancer cells and T cells.^[^ ^22^ ^]^ In here, hNP is consisted of three components: 1) PLGA polymeric core for hydrophobic drug loading and release, 2) biotin‐ and PEG‐ylated lipid layer to enhance cellular uptake and easy antibody modification, 3) sFVA moiety for AML cell‐targeting. To develop an HO1‐inhibitor‐loaded hNP, a PLGA‐polymeric core was complexed with various ratios of DSPE‐PEG_2000_ and DPPC (at a molar ratio of 1:3) as previously described.^[^ ^22b^ ^]^ The lipid weight ratio to PLGA of 0.25 indicated an increased *ζ*‐potential with −33.7 ± 2.71 mV and an average size of 162.9 ± 8.64 nm in comparison with −39.86 ± 2.85 mV and 198.5 ± 2.06 nm of PLGA nanoparticles (**Figure** **2**a). SnMP is an FDA‐approved HO1 inhibitor and has been used to treat hyper bilirubinemia.^[^ ^23^ ^]^ Among various SnMP to particle ratios, 4–6% were found to be the optimum loading amount without affecting the size and *ζ*‐potential of the prepared nanoparticles with 214.5 ± 0.7 nm in 6% drug amount loading (Figure 2b). As shown in Figure 2c, scanning electron microscopy imaging of prepared hNPs revealed that the PLGA particle is layered by a thin lipid membrane (indicated as an arrow). After preparation and concentration, the hNP retains its spherical shape, size, and poly dispersity index of 0.1–0.2 for more than a month. The SnMP‐loaded hNP is slightly larger than an empty hNP with 181 ± 3 and 144.1 ± 2.4 nm, respectively (Figure 2d). Finally, sFVA was complexed with hNP for binding on DSPE‐PEG_2000_‐biotin, with a weight to hNP ratio of 2.5–5% indicated as an optimal formulation (Figure 2e). In Figure 2f, the drug loading efficiency was 4.99 ± 0.15% and 4.98 ± 0.21% for 5.6% and 6.4% of initial drug loading, respectively, and which is converges to weight ratio 5% of hNP. The drug release study in Figure 2g shows 47.08 ± 5.45% of the drug was released from the hNP after 72 h at 37 °C.

### 2.3 Enhanced Cellular Uptake of Hybrid Nanoparticle in Leukemia Cells

**Figure 3:**
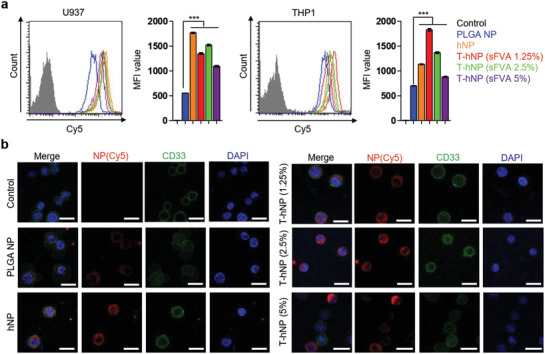
Enhanced cellular uptake of hybrid nanoparticle in leukemia cells. a) In vitro cellular uptake of Cy5‐loaded hybrid nanoparticle in leukemia cells. Each cell was incubated with PLGA and hybrid nanoparticles for 1hr and analyzed by flow cytometry (nanoparticle concentration: 5 µg mL^−1^). Data are presented as mean ± SD, and all statistical analyses were performed by one‐way ANOVA and Tukey's post‐hoc test, ***p < 0.001, *n* = 3 per group. b) Confocal microscopy image of cellular uptake of hybrid nanoparticles (Cy5, Red) in THP‐1 cells at 1hr after treatment of nanoparticles at a concentration of 5 µg mL^−1^. Cells were stained with anti‐CD33 antibody (green) for morphology imaging. Scale bar: 20 µm.

To evaluate enhanced cellular uptake by lipid‐layer and sFVA‐modification, THP‐1 and U937 cells were incubated with Cy5‐loaded nanoparticles and analyzed by flow cytometry. The size and *ζ*‐potential of Cy5‐loaded hNP were comparable with SnMP‐loaded hNP (Figure S1a, Supporting Information). In human AML cell lines (CD64+) THP‐1 and U937, hNPs showed 1.62‐and 3.2‐fold higher cellular uptakes in comparison with PLGA nanoparticles. However, sFVA‐modification on the surface of hNP at a weight ratio 1.25–5% exhibited different patterns in cellular uptake enhancements between two cell lines. In U937, sFVA‐modification reduced cellular uptake of hNP which differed from enhanced cellular uptake by 1.25–2.5% sFVA‐modification in THP‐1 cells, demonstrating different cellular uptake mechanism by lipid‐cell membrane interaction between these two cell lines (**Figure** **3**a). Confocal microscopy imaging showed similar Cy5 uptake patterns with flow cytometry data. In comparison with PLGA nanoparticle, hNPs with/without sFVA modification showed higher cellular uptake and were mostly distributed in cytoplasm upon surface binding and internalization (Figure 3b, Figure S1b, Supporting Information). Collectively, the hNP and sFVA‐modified hNP (T‐hNP) exhibited higher cellular uptakes than PLGA nanoparticles. Although higher sFVA modification hampered cellular internalization of hNP in vitro, targeted delivery with antibody was expected to represent more prominent effects in vivo. Therefore, 2.5% and 5% sFVA‐modification were chosen for in vivo study.

### 2.4 sFVA‐Mediated Bone Marrow Leukemia Cell Targeting and Biodistribution of Hybrid Nanoparticle in U937‐Bearing Orthotopic AML Model

**Figure 4:**
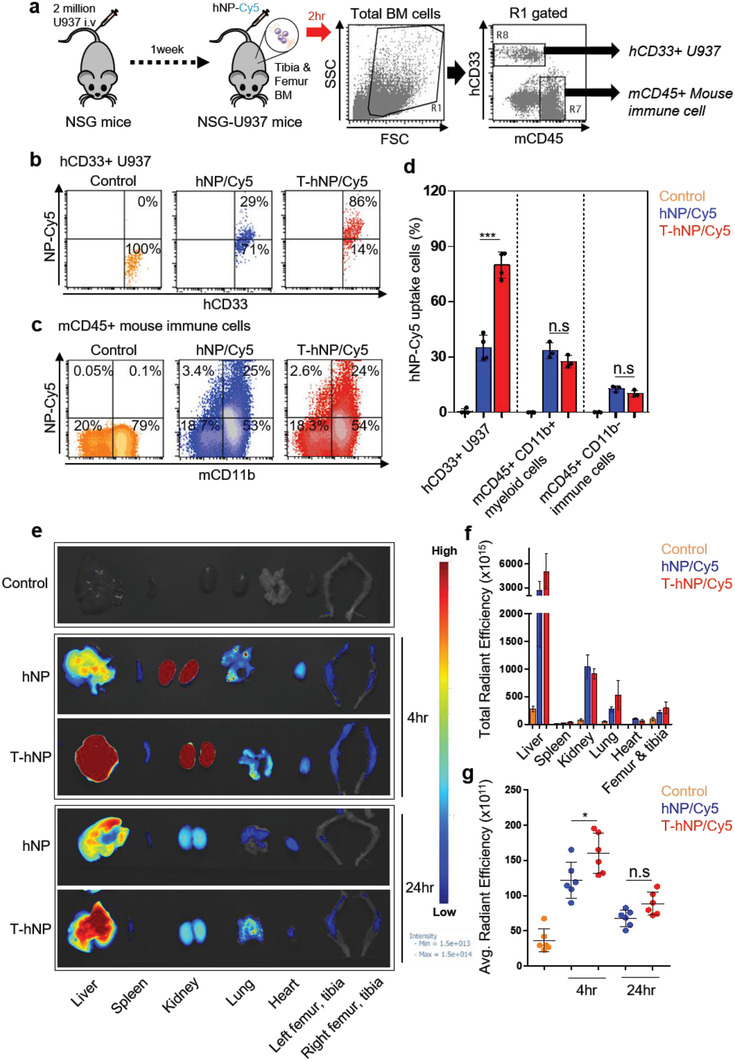
sFVA‐mediated bone marrow leukemia cell targeting and biodistribution of hybrid nanoparticle in U937‐bearing orthotopic AML model. a) Experimental procedures and FACS gating strategy for bone marrow cells. NSG mice 4–6 week old were infused with U937 cells and intravenously injected with hybrid nanoparticle at day 6 post cell infusion. At 2 h after nanoparticle injection, bone marrow cells were harvested from the femur and tibia and analyzed by flow cytometry. b) Representative dot plot of nanoparticle uptake for human CD33+ bone marrow U937 cells. c) Representative dot plot of nanoparticle uptake for mouse CD45+ CD11b+ bone marrow immune cells. d) Bar graph for the percentage of nanoparticle uptake for U937 and mouse immune cells. Data are presented as mean ± SD (*n* = 3–4 per group). e) Representative organ image for biodistribution of hNP and T‐hNP. f) Total radiant efficiency for organ distribution of nanoparticles at 24 h post injection. Total radiant efficiency for femur and tibia indicates sum of left and right femur and tibia and compared to other organs. g) Average radiant efficiency for nanoparticles in femur and tibia. Each dot indicates fluorescence intensity for left or right femur and tibia. Data are presented as mean ± SD (*n* = 3 per group). All statistical analyses were performed by one‐way ANOVA with Bonferroni's test, *p < 0.05, ***p < 0.001, n.s = not significant.

As previously reported,^[^ ^24^ ^]^ human leukemia xenograft has been developed with NOD‐SCID il2r gamma^−/−^ (NSG) mice deficient in T and B cell maturation and NK cell immune response. Despite of deficiency of adaptive immune response and gamma‐chain signaling, myeloid cells such as macrophage, monocyte, and neutrophil exist in NSG mice which enables to study innate immune‐cancer interaction and myeloid cell‐mediated immunotherapeutic effect.^[^ ^11^ ^]^ The CD64+ CD33+ U937 cells were injected intravenously into NSG mice and modeling was validated as described in our previous study (Figure S2, Supporting Information).^[^ ^15^ ^]^ Human U937 cells are commonly accumulated in liver and bone marrow niches followed by enlarged spleens which recapitulate human AML pathologies.^[^ ^25^ ^]^ Bone marrow is a clinically relevant, dominating organ in blood cancers,^[^ ^26^ ^]^ and leukemia‐targeted delivery was evaluated in bone marrow. The hNP and sFVA‐modified T‐hNP were injected into an orthotopic AML model and their uptake into bone marrow leukemia cells was analyzed from the tibia and femur by using flow cytometry (**Figure** **4**a). As shown in Figure 4b, human CD64+ CD33+ U937 cells showed cellular uptake of 79.8 ± 7.2% for T‐hNP (5% sFVA) and 35 ± 6.9% for hNP. In addition, sFVA‐modification at 5% resulted in higher leukemia cell‐targeted uptake than 2.5% (Figure S3, Supporting Information). In Figure 4c, hNP was shown to be internalized by 33.5 ± 4.3% of mouse CD11b+ bone marrow myeloid cells and T‐hNP showed a slightly reduced uptake by 27.5 ± 3.3%, which confirmed that sFVA‐modification enhanced leukemia cell‐targeted uptake of hNP. It should be pointed out that only 10.1 ± 1.7% of the CD11b‐ immune cells internalized T‐hNP (Figure 4d). Macrophages and monocytes are mononuclear phagocytes naturally engulfing nanoparticles more than other cell types.^[^ ^27^ ^]^ In a previous study, the negatively charged surface of nanoparticles was shown to enhance phagocytic‐ and myeloid‐cell uptake.^[^ ^28^ ^]^ At 10 days post cell infusion, orthotopic AML xenografts were intravenously injected with Cy5‐loaded hNP and T‐hNP. Major organs and femur and tibia were harvested to measure fluorescence intensity. Both hNP and T‐hNP highly localized to liver and kidney which are major clearance routes for nanoparticles (Figure 4e). The hNP and T‐hNP localization in femur and tibia was quantified and compared with other organs. In comparison with hNP, T‐hNP showed higher accumulation in liver, lung, and femur and tibia, which are attributable to leukemia‐enriched organ targeting effects (Figure 4f). As previously described, liver and bone marrow are major U937 accumulation organ and lung is also a probable organ due to the size of cells.^[^ ^29^ ^]^ Average radiant efficiency analysis in femur and tibia of T‐hNP group showed 1.3‐fold higher intensity compared to hNP group which is reasonable to explain bone marrow leukemia‐targeted delivery by sFVA‐modification (Figure 4g). Our previous study showed that U937 cells comprise 10% to 25% of bone marrow cell at 10 days post AML modeling. Collectively, sFVA‐modification enhanced active targeting of nanoparticles to CD64+ leukemia cells in bone marrow and leukemia niche organs, and passively targeting to CD11b+ myeloid cells.

### 2.5 In Vitro Chemo‐Sensitization Effect of HO1‐Inhibiting Hybrid Nanoparticle in Leukemia Cells

**Figure 5:**
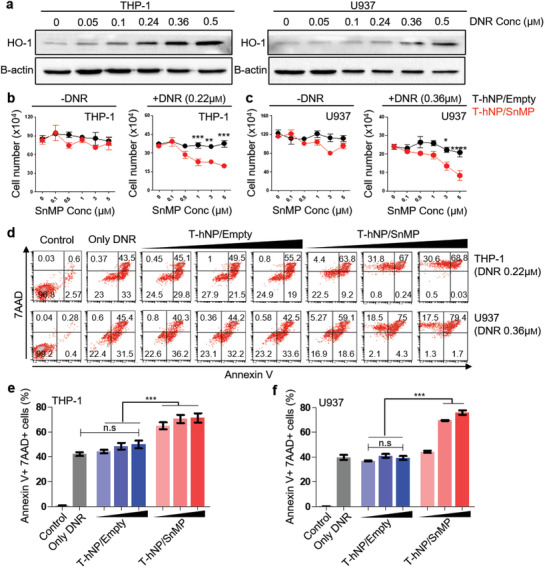
In vitro chemo‐sensitization effect of HO1‐inhibiting hybrid nanoparticle in leukemia cells. a) Western blot image of DNR‐responsive HO1 overexpression in leukemia cells. The HO1 protein was detected in THP‐1 and U937 cells 24 h after exposure to various concentrations of DNR. b) Cell viability test in THP‐1 leukemia cells. c) Cell viability test in U937 leukemia cells. Cell viability was measured after 24 h of DNR and nanoparticle treatment. Data are presented as mean ± SEM, and all statistical analyses were performed by two‐way ANOVA and Tukey's post‐hoc test, *p < 0.05, **p < 0.01, ***p < 0.001, *n* = 3 per group for ‐DNR, *n* = 5–7 per group for +DNR. d) Apoptosis assay for chemo‐sensitization by T‐hNP/SnMP in leukemia cells. Annexin V and 7AAD were stained and analyzed by flow cytometry at 30 h after DNR and nanoparticle treatment. e,f) Bar graph of apoptosis assay in (d). Data are presented as mean ± SD, ***p < 0.001 by one‐way ANOVA with Tukey's post‐hoc test, n.s = not significant (*n* = 3 per group).

Previous studies have demonstrated chemo‐resistant effect of HO1 in various cancers and AML.^[^ ^13^, ^14^ ^]^ Recently, we have reported that siRNA‐mediated HO1 inhibition enhances chemo‐sensitivity in an AML xenograft model and patient‐derived cells.^[^ ^15^ ^]^ To evaluate the chemo‐sensitization effect of HO1‐inhibiting hNPs, THP‐1, and U937 cells were treated with empty‐ and SnMP‐loaded T‐hNPs in the presence of daunorubicin (DNR), a first‐line chemotherapeutic for AML. The HO1 was overexpressed depending on the concentration of DNR in the THP‐1 and U937 cells (**Figure** **5**a). In Figure 5b,c, T‐hNP/SnMP improved the cytotoxic effect of DNR at SnMP concentrations of 1 to 5 µм. However, no cytotoxic effects were observed in the absence of DNR. Flow cytometry data revealed increased apoptotic responses of leukemia cells to DNR at various concentrations of T‐hNP/SnMP compared to T‐hNP/Empty group. (Figure 5d–f).

### 2.6 Combination Therapy of HO1‐Inhibiting T‐hNP with Daunorubicin Suppresses Leukemia Growth in Human AML‐Bearing Orthotopic Model

**Figure 6:**
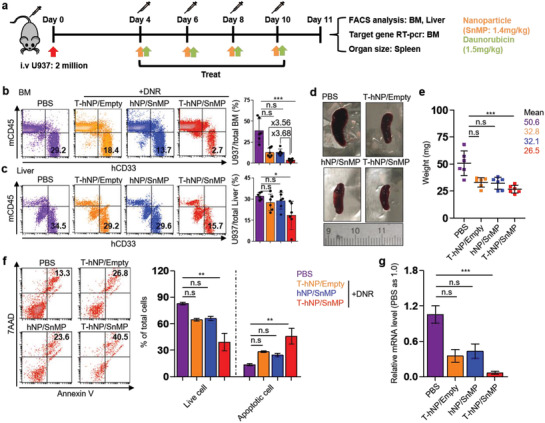
Combination therapy of HO1‐inhibiting T‐hNP with daunorubicin suppresses leukemia growth in human AML‐bearing orthotopic model. a) Experimental schedule for an in vivo therapeutic study. The 5–6 week old NSG mice were intravenously injected with 2 million U937 cells and treated 4, 6, 8, and 10 days after cell infusion, and their organs were harvested at day 11 for analysis. DNR was injected intravenously after 6 h of nanoparticle treatment. b) Leukemia growth in bone marrow. Total bone marrow cells from tibia and femur were stained with human CD33 mAb and mouse CD45 mAb for flow cytometric analysis. c) Leukemia growth in liver. Total liver cells were homogenized and stained for analysis. d) A representative spleen images. e) Bar graph for spleen weight. Data are presented as mean ± SD, *p < 0.05, ***p < 0.001 by non‐parametric Kruskal–Wallis‐test, n.s = not significant (*n* = 6 per group for b, c, e). f) Apoptosis assay for human CD33+ U937 cell in bone marrow. Total bone marrow cells were harvested and stained for gating of CD33+ leukemia cell. The bar graph indicates live cells (Annexin V‐, 7AAD‐) and apoptotic cells (Annexin V+). g) qRT‐PCR analysis. The mRNA level of human GAPDH was measured in total bone marrow cells and normalized by mouse GAPDH levels. Data are presented as mean ± S.E.M, **p < 0.01, ***p < 0.001 by non‐parametric Kruskal–Wallis‐test, n.s = not significant (*n* = 3–4 per group for (f), *n* = 5–6 per group for (g)).

A human U937 AML xenograft model has been used to distinguish mouse myeloid cells from human cells, which facilitated experimental analysis of immune reprogramming in bone marrow niche myeloid cells. Recent study showed that HO1 acted as an immune checkpoint molecule in myeloid cell and a combination therapy of SnMP with 5‐FU boosted anti‐tumor immune response in breast tumor model.^[^ ^17^ ^]^ As previously described before,^[^ ^17^ ^]^ many kinds of chemotherapeutics induce anti‐cancer immune responses. Additionally, most of immunotherapeutic reagents show outstanding anti‐tumor effect when only it combined with chemotherapeutic and other immunotherapeutic.^[^ ^17^, ^30^ ^]^ Based on the chemo‐sensitization effect and immune checkpoint function of HO1,^[^ ^17^ ^]^ T‐hNP/SnMP was combined with DNR in human AML‐bearing orthotopic model. Empty T‐hNP +DNR group represents chemotherapy by DNR and T‐hNP/SnMP +DNR group represents chemo‐and immuno‐combination therapy. First, the anti‐cancer effect of HO1‐inhibiting T‐hNP was evaluated in an orthotopic AML model. Xenograft mice were injected 4 times with nanoparticles and treated with DNR, and their organs were analyzed at day 11 (**Figure** **6**a). As shown in Figure 6b, leukemia cell growth in bone marrow was significantly reduced in the T‐hNP/SnMP +DNR treatment group, at a rate that was 3.68‐fold and 3.56‐fold lower than with hNP/SnMP +DNR and T‐hNP/Empty +DNR groups, respectively. The T‐hNP/SnMP +DNR also suppressed growth of liver‐enriched leukemia cells (Figure 6c). In Figure 6d,e, the T‐hNP/SnMP +DNR group showed highly reduced splenomegaly. Furthermore, 1.63‐fold and 1.87‐fold more apoptotic CD33+ U937 cell populations were detected in the bone marrow of the T‐hNP/SnMP +DNR treatment group in comparison with T‐hNP/Empty and hNP/SnMP groups, respectively (Figure 6f). In Figure 6g, less amount of human GAPDH mRNA was measured in the bone marrow of the treatment group, which is consistent with the flow cytometry results of Figure 6b.

### 2.7 Immune Reprogramming and Activation Effect of HO1‐Inhibiting T‐hNP in Bone Marrow Myeloid Cells

**Figure 7:**
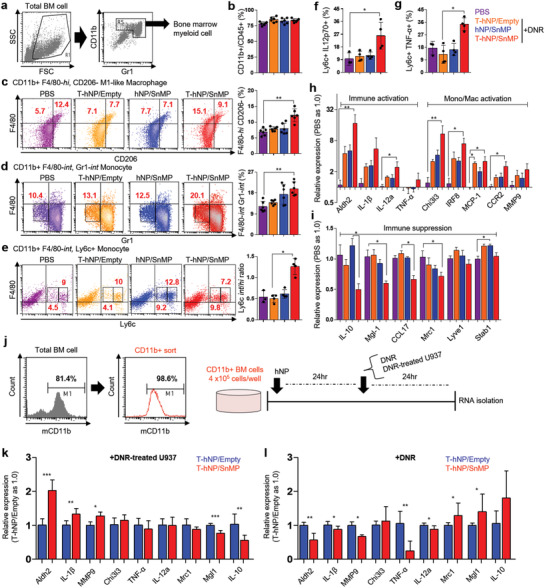
Immune reprogramming and activation effect of HO1‐inhibiting T‐hNP in bone marrow myeloid cells. a) Gating strategy for bone marrow myeloid cells. The CD11b+ and Gr1+ cells were gated for further analysis. b) The ratio of CD11b+ myeloid cells to CD45+ total immune cells. c) The ratio of F4/80‐hi, CD206‐ M1‐like macrophages in total myeloid cells. d) The ratio of Gr1‐int and F4/80‐int monocytic cells in total myeloid cells. e) The ratio of Ly6c‐int and Ly6c‐hi monocyte in total bone marrow myeloid cells. f) Flow cytometric analysis of intracellular IL‐12p70 expression in bone marrow CD11b+ Ly6c+ monocytes. g) Flow cytometric analysis of intracellular TNF‐*α* expression in bone marrow CD11b+ Ly6c+ monocytes. Data are presented as mean ± SD, *p < 0.05, **p < 0.01 by a non‐parametric Kruskal–Wallis‐test, n.s = not significant (*n* = 6 per group for b, c, d, *n* = 3–4 per group for e, f, and g). h) Immune activation and monocyte/macrophage activation marker gene expression levels in bone marrow. i) Immune suppression and M2‐like macrophage marker gene expression levels in bone marrow. Data are presented as mean ± SEM, *p < 0.05, **p < 0.01 by non‐parametric Kruskal–Wallis‐test, n.s = not significant, *n* = 5 per group for h and i. j) Magnetic cell sorting for CD11b+ myeloid cells and experimental scheme for ex vivo analysis. Bone marrow cells were harvested from C57BL/6 mice and analyzed by flow cytometry before and after sorting. k) Marker gene expression levels in HO1‐inhibited CD11b+ myeloid cells in response to apoptotic leukemia. l) Marker gene expression levels in HO1‐inhibited CD11b+ myeloid cells in response to chemotherapy. Data are presented as mean ± SD, *p < 0.05, **p < 0.01, ***p < 0.001 by Student's *t*‐test, n.s = not significant, *n* = 3–4 per group for (k,l).

To validate the immune reprogramming and activation effects of HO1‐inhibiting T‐hNP, mouse bone marrow myeloid cells were analyzed by flow cytometry. As shown in **Figure** **7**a, CD11b+ cells were gated as total bone marrow myeloid lineages. The total CD11b+ myeloid cell % to CD45+ immune cell did not change significantly between groups (Figure 7b). The F4/80‐hi CD206‐ M1‐like and F4/80‐hi CD206+ M2‐like macrophages were analyzed,^[^ ^31^ ^]^ and CD206‐ M1‐like cells were increased in T‐hNP/SnMP group with 12.1 ± 2% compared to 7.83 ± 0.66% and 7.9 ± 1.7% in T‐hNP/Empty and hNP/SnMP, respectively (Figure 7c). However, F4/80‐hi CD206+ M2‐like macrophage was not significantly reduced (Figure S4a, Supporting Information). The M1/M2 ratio of T‐hNP/SnMP +DNR group was also higher than other groups (Figure S4b, Supporting Information). Gr1‐intermediate (Gr1‐int) and F4/80‐intermediate (F4/80‐int) myeloid cells were increased in the hNP/SnMP +DNR and T‐hNP/SnMP +DNR groups with 17.3 ± 4.2% and 19.8 ± 2.9%, respectively (Figure 7d). Gr1 is Ly6c/Ly6G and Gr1‐int, F4/80‐int cells are generally monocytic lineages,^[^ ^32^ ^]^ and CJ Perry et al. demonstrated Chi3l3+ Ly6c+ F4/80‐int monocyte attraction in melanomas after myeloid‐targeted immunotherapy, which is a polyfunctional inflammatory cell with increased cytokine expression.^[^ ^33^ ^]^ Total Ly6c+ monocytic cell % was not significantly different between groups (Figure S4c, Supporting Information). However, the ratio of Ly6c‐int to Ly6c‐hi monocytes was increased in T‐hNP/SnMP +DNR group of 1.26 ± 0.1 in comparison with 0.5 ± 0.09 and 0.6 ± 0.1 of T‐hNP/Empty +DNR and hNP/SnMP +DNR, respectively (Figure 7e). These results demonstrate Ly6c‐int monocyte recruitment and phenotypic change of monocyte population. Furthermore, in Figure 7f,g, 2.2‐fold and 2.3‐fold higher % of CD11b+ Ly6c+ monocytes from T‐hNP/SnMP +DNR expressed intracellular levels of inflammatory cytokines, IL12p70 and TNF‐*α*, respectively. Non‐monocytic Ly6c‐ CD11b+ cells show less prominent upregulation of intracellular cytokine (Figure S4d, Supporting Information). Immune activation, suppression, and monocyte/macrophage gene expressions were analyzed in bone marrow cells to evaluate the immune reprogramming effect of T‐hNP/SnMP treatment. In Figure 7h, immune activation‐relevant genes such as IL‐12a, IL‐1*β*, and Aldh2 were mostly upregulated in T‐hNP/SnMP +DNR in comparison with other groups, and monocyte/macrophage activation markers such as interferon regulatory factor 8 (IRF8) and CCR2 were increased, which was consistent with the result of monocyte/macrophage phenotype change in flow cytometric analysis. In previous studies, IRF8 activation demonstrated immunotherapeutic effect and human leukemia inhibition.^[^ ^8^, ^34^ ^]^ The increased level of Chi3l3 was reasonable to explain by Chi3l3+ Ly6c+ polyfunctional monocyte attraction.^[^ ^33^ ^]^ M2‐like macrophage and immune suppression‐relevant gene expressions showed decreased levels of IL‐10, Mgl‐1, and Mrc1 (CD206) in the T‐hNP/SnMP +DNR treatment group (Figure 7i). IL‐10 is a major immune‐suppressive cytokine and Mgl‐1 is a C‐type lectin receptor for glycan and related with tumor‐associated macrophage and immune suppression.^[^ ^35^ ^]^ A reduced chemokine, CCL17 was associated with unfavorable prognoses of tumors and attraction of regulatory T cells in a previous study.^[^ ^36^ ^]^ In comparison with T‐hNP/SnMP, only a modest change in gene expression was measured in the hNP/SnMP group even comparable Gr1‐int, F4/80‐int monocytic cell recruitment (Figure 7d). As an immune checkpoint molecule in myeloid cells,^[^ ^17^ ^]^ HO1‐inhibition shows therapeutic effect only when it combined with chemotherapeutics suggesting that chemo‐induced specific conditions trigger HO1‐inhibition‐mediated immune activation. To understand the improved anti‐leukemic and immune activation mechanism of the T‐hNP/SnMP group, CD11b+ bone marrow cells were sorted by magnetic beads and analyzed ex vivo (Figure 7j). The magnetic sorted CD11b+ cell showed ≈98.6% purity and exposed to DNR or DNR‐treated U937 at 24 h after T‐hNP treatment, could discriminate immune activation response to DNR from response to apoptotic leukemia cells (DNR‐treated U937). As shown in Figure 7k,l, T‐hNP/SnMP treatment modulates expression levels of listed‐genes as comparable to in vivo analysis. The T‐hNP/SnMP treated myeloid cells responded strongly to apoptotic leukemia cells with increased inflammatory genes and reduced immune suppressive gene expression. Interestingly, HO1‐inhibition‐mediated gene expression was reversed in response to DNR treatment. Collectively, HO1‐inhibiting T‐hNP reprogrammed bone marrow myeloid cells by recruiting Gr1‐int, Ly6c‐int, F4/80‐int monocytic cells, inducing F4/80‐hi, CD206‐ M1‐like macrophages, consequently, enhances the immune activation response against apoptotic leukemia cells. In comparison with hNP/SnMP treatment, increased leukemic apoptosis in T‐hNP/SnMP is a condition for immune boosting effect in HO1 checkpoint‐inhibited myeloid cell.

### 2.8 Survival Study and Therapeutic Mechanisms of Chemo‐ and Immuno‐Combination Therapy by HO1‐Inhibiting T‐hNP

**Figure 8:**
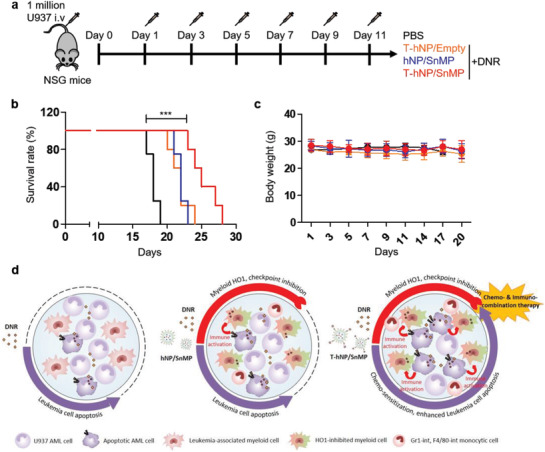
Survival study and therapeutic mechanisms of chemo‐ and immuno‐combination therapy by HO1‐inhibiting T‐hNP. a) Experimental schedule for survival study (SnMP dose: 1.4 mg kg^−1^, DNR dose: 1.5 mg kg^−1^ at days 1, 3, 5, 7, 9, and 11). b) Survival study (*n* = 4–5 per group). c) Body weight graph. Data is presented as mean ± SD, *n* = 4–5 per group. d) Experimental summary of therapeutic mechanism for HO1‐inhibiting T‐hNP.

Finally, in vivo therapeutic benefit with respect to survival was validated in an orthotopic AML model. A human AML‐bearing xenograft model was injected with nanoparticles and DNR 6 times after cell infusion and its survival and body weight was monitored (**Figure** **8**a). The overall survival was 19, 24, 23, and 28 days for PBS, T‐hNP +DNR, hNP/SnMP +DNR, and T‐hNP/SnMP +DNR, respectively (Figure 8b,c). Only moderate survival prolongation was demonstrated which is attributable to adaptive T cell immunity deficiency in NSG mice which is a key factor affecting cancer immunotherapy efficiency.^[^ ^17^, ^33^ ^]^ Collectively, our study demonstrates HO1 inhibitor‐mediated chemo‐sensitization in leukemia cells and HO1 inhibitor‐mediated immune activation of myeloid cells as mechanisms for a combination therapy of T‐hNP/SnMP with DNR (Figure 8d).

## 3 Discussion

In recent studies of AML therapy, multiple leukemic clones have been detected that induces a relapse and de novo leukemia genesis and therapy resistance and complicates leukemia treatment. Meanwhile, targeting environmental cells in a leukemia niche such as a macrophage/monocyte is a promising strategy due to its prevalence in various kinds of tumors.^[^ ^7^ ^]^ CD64 expressions in leukemia and tumor macrophages has been reported,^[^ ^15^, ^37^ ^]^ which supports clinical relevance of CD64‐targeting. Previously, HO1 siRNA‐mediated chemo‐sensitization has been demonstrated in an AML xenograft model and AML patient bone marrow samples.^[^ ^15^ ^]^ Here, we evaluated dual cell‐targeted HO1‐inhibition for a synergistic effect of chemo‐sensitization and immune reprogramming by using HO1 inhibitor‐loaded lipid‐polymer hNP. T‐hNP was actively targeted to CD64+ leukemia cells by engineered antibody moiety and passively targeted to CD11b+ myeloid cells through their phagocytic nature and negatively charged surface. The T‐hNP/SnMP treatment enhanced DNR‐responsive apoptosis in leukemia cells and activates immune responses in myeloid cells against apoptotic leukemia. Although NSG mice present an appropriate model for studying human leukemia xenograft, it is difficult to evaluate anti‐tumor immune generation due to a lack of T and B cell immune maturation and NK cell immunity. Our results demonstrate immunotherapeutic effects by innate myeloid cell immune response, not by adaptive anti‐tumor immune response that is attributable to the moderate effects of T‐hNP/SnMP +DNR treatment in survival study. The SnMP‐mediated HO1‐inhibition in myeloid cells induces an anti‐tumor immune response via CD8+ T cell adaptive immune responses,^[^ ^17^ ^]^ and the therapeutic effects of HO1‐inhibiting T‐hNP should be evaluated in an AML patient‐derived xenograft model bearing a human immune system.^[^ ^38^ ^]^ As described before,^[^ ^17^ ^]^ HO1 is an enzyme breaking down dying cell released heme, consequently producing biliverdin, Fe2+ and carbon monoxide (CO). The CO is closely related with p38 MAPK, STAT1/3, and NFĸB signaling, which have been proposed for HO1‐mediated cytoprotective and anti‐inflammatory effects. However, exact molecular mechanism has not yet understood how HO1 acts on myeloid cell reprogramming and macrophage polarization, and molecular mechanisms of HO1‐mediated myeloid cell reprogramming and polarization should be studied in the future. Various chemotherapeutic drugs other than DNR should be combined with an HO1‐inhibiting strategy to optimize immunotherapeutic effects. Cytarabine, a pyrimidine analog similar to 5‐fluorouracil, is a promising candidate.^[^ ^17^ ^]^ For antibody fusion proteins, simple modification using an engineered antibody is a promising targeted delivery strategy with a broad spectrum of potential applications, from cancers to inflammatory diseases.

## 4 Conclusion

HO1‐inhibiting lipid‐polymer hybrid nanoparticle (T‐hNP) actively targeted to human leukemia cells by engineered antibody and passively targeted to CD11b+ myeloid cells in a bone marrow niche of human AML‐bearing orthotopic mouse model. T‐hNP‐mediated HO1‐inhibition enhanced the chemo‐therapeutic effect of DNR and boosted immune response by reprogramming bone marrow myeloid cells. HO1‐inhibitng dual cell‐targeted hNP with DNR has a strong potential as a novel therapeutic in AML by providing chemo‐sensitization of AML cells and immune activation of bone marrow myeloid cells.

## 5 Experimental Section

##### Materials

SnMP, DNR hydrochloride, poly(lactic‐*co*‐glycolic acid) (PLGA, lactide: glycolide 50:50, 7000–17000 Da), and biotin‐FITC were purchased from Sigma Aldrich (St. Louis, MO, USA). 1,2‐Dipalmitoyl‐sn‐glycero‐3‐phosphocholine (DPPC) and 1,2‐distearoyl‐sn‐glycero‐3‐phosphoethanolamine‐*N*‐[biotinyl(polyethylene glycol)‐2000] (DSPE‐PEG_2000_‐Biotin), 1,2‐distearoyl‐sn‐glycero‐3‐phosphoethanolamine‐*N*‐[amino(polyethylene glycol)‐2000] (DSPE‐PEG_2000_) were obtained from Avanti Polar Lipids, Inc. (Alabaster, AL, USA). Anti‐human CD33, CD64 antibodies and anti‐mouse CD11b, CD45, CD206, Ly6c, Gr1, TNF‐*α*, IL12p70, Rat IgG1 Isotype antibodies were purchased from BD Biosciences (USA) (Table 1, Supporting Information). Anti‐mouse F4/80 antibody and Avidin‐FITC were purchased from Biolegend (San Diego, CA, USA). Anti‐human CCR2 antibody was obtained from R&D Systems (Minneapolis, MN, USA). Anti‐His‐Tag, anti‐human HO1 (P249), and *β*‐actin antibodies (13E5) were obtained from Cell Signaling Technology (Danvers MA, USA).

##### Vector Construction

A 429 and 888 base pair sequence for monomeric avidin and anti‐CD64 scFv were cloned (Incorporation Bioneer, Korea) in pET21a (Novagen, Madison, WI) by NotI, xhoI and xbaI, NotI sites, respectively, for bacterial expression.

##### Hybrid Nanoparticle Preparation

DSPE‐PEG_2000_ (ratio of 5:1 for biotinylated to non‐biotinylated) and DPPC were mixed at a molar ratio of 1:3 and stored for 1 h at room temperature to evaporate the chloroform. The prepared lipid mixture was hydrated in water (4% EtOH, 10 mL) at 0.2 mg mL^−1^ and gently stirred. SnMP (400 µg) and PLGA (7.2 mg) solutions were prepared at concentrations of 4 mg mL^−1^ in dimethyl sulfoxide (DMSO) and 2.4 mg mL^−1^ in dichloromethane, respectively. The drug/PLGA solution (836 µL) was dropped slowly to a lipid solution (2.4 mL) at a ratio of 1:3 (v/v, PLGA: lipid), sonicated and evaporated to remove the dichloromethane. The prepared particle solution (1 mg mL^−1^) was concentrated and washed through a cellulose membrane (MWCO 30 000 Da) at 2.5–10 mg mL^−1^.

##### sFVA Protein Expression and Purification

BL21 (DE3) cells (Novagen, Madison, WI) were transformed with a sFVA‐cloned pET21a vector and cultured in 20 mL of Amp+ lysogeny broth (LB) at 37 °C. After 2–4 h of incubation, the cells were cultured in 0.5 L of LB medium. When the optical density at 600 nm reached 0.2–0.3, 1 mm isopropyl *β*‐D‐1‐thiogalactopyranoside (IPTG) was added and the cells were induced for 4 h at 37 °C. The induced pellet was re‐suspended in a lysis buffer (pH 8.0) and then sonicated (pulse on: 20 s, total 2 min, off: 59 s, amplitude: 30%). The protein solution was then collected through centrifugation at 27 500 g, and the resulting solution was filtered using a 0.45 µm filter.

##### Affinity Chromatography Purification

The protein solution was loaded to a Ni‐NTA agarose resin (Qiagen)‐charged column and washed with 40 volume equivalents of washing buffer. The resin‐bound protein was eluted at 250 mm imidazole elution buffer. The purified protein was dialyzed using a Slide‐A‐Lyzer Dialysis cassettes (Thermo Fisher Scientific, 12 mL, CA; MWCO 10 000 Da) in presence of a refolding buffer (pH 8.2) and dialyzed through a phosphate buffered saline (PBS) at pH of 7.4. Protein was concentrated through a cellulose membrane (MWCO 10 000 Da).

##### Cell Culture

Human THP‐1 and U937 leukemia cells were purchased from ATCC (Virginia, USA) and cultured at 37 °C in 5% CO2 in RPMI1640 medium (Welgene, Korea) supplemented with 10% fetal bovine serum and 1% penicillin. Cells were passaged to a density of 1–2 × 10^5^ cells mL^−1^ and media was changed every 2–3 days.

##### SDS‐PAGE and Western Blotting

Purified and PBS‐dialyzed protein was mixed with Laemmli buffer (5 mm dithiothreitol), boiled for 15 min and loaded into 12% SDS‐PAGE gels for electrophoresis. Gel was stained with Coomassie blue or transferred to a polyvinylidene fluoride membrane (Millipore, Billerica, MA) for immunodetection by anti‐His‐Tag antibody (Cell Signaling Technology, Danvers MA, USA) and anti‐rabbit IgG antibody‐HRP (Santa Cruz, Texas, DA, USA).

##### Competitive Binding Study

THP‐1 cells (2 × 10^5^ cells well^−1^) were incubated with anti‐CD64 mAb‐FITC (BD Pharmingen) in the presence of sFVA (10 µg mL^−1^) in PBS at 4 °C for 20 min. After the cells were washed twice, they were analyzed using FACSCalibur (BD Biosciences, USA). For biotin‐competitive binding of sFVA, the THP‐1 cells were incubated with anti‐CCR2 mAb‐biotin in the presence of sFVA and avidin‐FITC.

##### Drug Loading Efficiency and Release Profiling

After preparation of SnMP‐loaded hNPs (10 mg mL^−1^), 0.6–0.7 mg particles were used to measure the loading efficiency and encapsulation efficiency at absorbance 399 nm using Tecan. The 10 mg mL^−1^ particle was resuspended in PBS (DMSO 10%) and centrifuged to harvest the released medium at 6, 12, 24, 48, and 72 h. Release media and particle were freeze‐dried and resuspended for detection of SnMP at absorbance 399 nm.

##### Characterization of Hybrid Nanoparticles

The prepared particles (1 mg mL^−1^) were diluted in water and analyzed with Zeta‐Sizer (Malvern) to optimize lipid/PLGA, particle/drug, and particle/sFVA ratios. The size of the hybrid particles (10 mg mL^−1^) was measured at indicated days and weeks after preparation to evaluate stability.

##### Cellular Uptake and Confocal Microscopy Imaging

THP‐1 and U937 cells (1 × 10^6^ cells mL^−1^) were incubated with Cy5‐loaded PLGA and hNPs at a concentration of 5 µg mL^−1^ for 1 h and analyzed by flow cytometry. Cells were stained with DAPI Fluoromount‐G (Southern Biotech) and imaged by confocal microscopy (Leica).

##### Daunorubicin‐Responsive HO1 Upregulation in Leukemia Cells

THP‐1 and U937 cells (4 × 10^5^ cells mL^−1^) were seeded and cultured in complete medium with various concentrations of DNR for 24 h. The cells were lysed using RIPA buffer and total protein was used for immunodetection by using anti‐human HO1 and *β*‐actin antibodies (Cell Signaling Technology, Danvers, MA, USA).

##### Cell Viability Test and Apoptosis Assay

Seeded THP‐1 and U937 cells (4–5 × 10^5^ cells mL^−1^, 24 well plate) were treated with hNPs (SnMP concentration: 1, 3, 5 µм). 5 h after treatment, DNR was added and incubated for an additional 24 and 30 h for further analysis. Total cell numbers were calculated using a hemocytometer. For apoptosis assay, cells were stained with Annexin V and 7AAD (BD Biosciences, USA) and analyzed by flow cytometry.

##### Orthotopic Acute Myelogenous Leukemia Modeling

4–6 week‐old male NOD‐SCID il2r gamma^−/−^ (NSG) mice (Jackson Laboratory) were intravenously injected with 1–2 × 10^6^ U937 cells and their survival was evaluated under SPF conditions. All animal experimental procedures were reviewed and approved by the Institutional Animal Care and Use Committee (IACUC) of Hanyang University (2019‐0076A) and were performed in accordance with the relevant guidelines.

##### In Vivo Leukemia‐Targeted Delivery of Hybrid Nanoparticle

1 week post cell infusion, U937‐bearing NSG mice were intravenously injected with hNP and T‐hNP (Cy5, 0.6 mg kg^−1^) and after 2 h, bone marrow cells were harvested from the femur and tibia and filtered through a 100 µm filter. Red blood cells were lysed and stained with anti‐human CD33, mouse CD11b, and CD45 antibodies for flow cytometric analysis.

##### In Vivo Biodistribution of Hybrid Nanoparticle

At day 10 post U937 cell infusion of NOD‐SCID il2r gamma^−/−^ mice, hNPs were intravenously injected (Cy5, 0.6 mg kg^−1^). The mice were sacrificed and Cy5 fluorescence intensity was measured in major organs at 4 and 24 h post injection using VISQUE InVivo Smart (Vieworks Co, Korea) in the Korea Basic Science Institute (Chuncheon, Korea).

##### In Vivo Therapeutic Study in an Orthotopic Model

U937‐bearing NSG mice were intravenously injected with hNPs (SnMP dose: 1.4 mg kg^−1^) at 4, 6, 8, 10 days post cell infusion. At day11, major leukemia niche organs were harvested and analyzed for further experimental analysis.

##### Bone Marrow Myeloid Cell Analysis and Gene Expression

After treatment, total bone marrow cells were harvested from femur and tibia and filtered through a 100 µm filter. Red blood cells were lysed and stained with myeloid cell lineage markers for flow cytometry analysis. Total RNA was isolated from bone marrow cells and reverse transcribed to cDNA using iScript cDNA synthesis kit (Bio‐Rad) to measure marker gene expression levels. All primers were synthesized and purchased from IDT DNA.

##### Ex Vivo Myeloid Cell Reprogramming

Total bone marrow cells were isolated from 5–7 week‐old C57BL/6 mice (Orient Bio) and sorted using magnetic EasySep Mouse CD11b positive Selection Kit (STEMCELL Technologies, USA). Purity was validated by FACSCalibur (BD Biosciences, USA) and seeded (4 × 10^5^ cells mL^−1^). 24 h post treatment of hNP, DNR, and DNR‐exposed U937 cells (DNR: 0.2 µм) were added. Total RNA was isolated, and reverse transcribed to cDNA (iScript cDNA synthesis kit, Bio‐Rad) and gene expressions were measured. For DNR‐exposed U937, U937 cells were exposed to DNR for 5 h and washed twice and added to myeloid cells (8 × 10^4^ cells mL^−1^).

##### In Vivo Therapeutic and Survival Study

4–6 week‐old NSG mice were injected with 1 × 10^6^ U937 cells intravenously via tail vein injection. At days 1, 3, 5 7, 9, and 11 post cell infusion, hNPs (SnMP: 1.4 mg kg^−1^) and DNR (1.5 mg kg^−1^) were intravenously injected and further monitored for survival rate.

##### Statistical Analysis

All data are presented as mean ±SD and SEM. Statistical analyses were performed using a Student's *t*‐test and one‐way ANOVA with Tukey's post‐hoc test in GraphPad Prism 7 Project software. All animal studies were analyzed using a non‐parametric Kruskal–Wallis test.

## Conflict of Interest

The authors declare no conflict of interest.

## Supporting information

Supporting Information
