# Supplementary material for: Heme Oxygenase 1‐Targeted Hybrid Nanoparticle for Chemo‐ and Immuno‐Combination Therapy in Acute Myelogenous Leukemia
Source: Adv Sci (Weinh). 2020 Jun 3;7(13):2000487. doi: 10.1002/advs.202000487 (PMC7341080; doi:10.1002/advs.202000487)
Supplement: Supplementary file 1 — Supporting Information [file ADVS-7-2000487-s001.pdf]

Supporting Information

Heme Oxygenase 1-targeted Hybrid Nanoparticle for Chemo- and Immuno- Combination Therapy in Acute Myelogenous Leukemia

Seok-Beom Yong, Jaehyun Kim, Jee Young Chung, Sehee Ra, Seong Su Kim,

*Yong-Hee Kim**

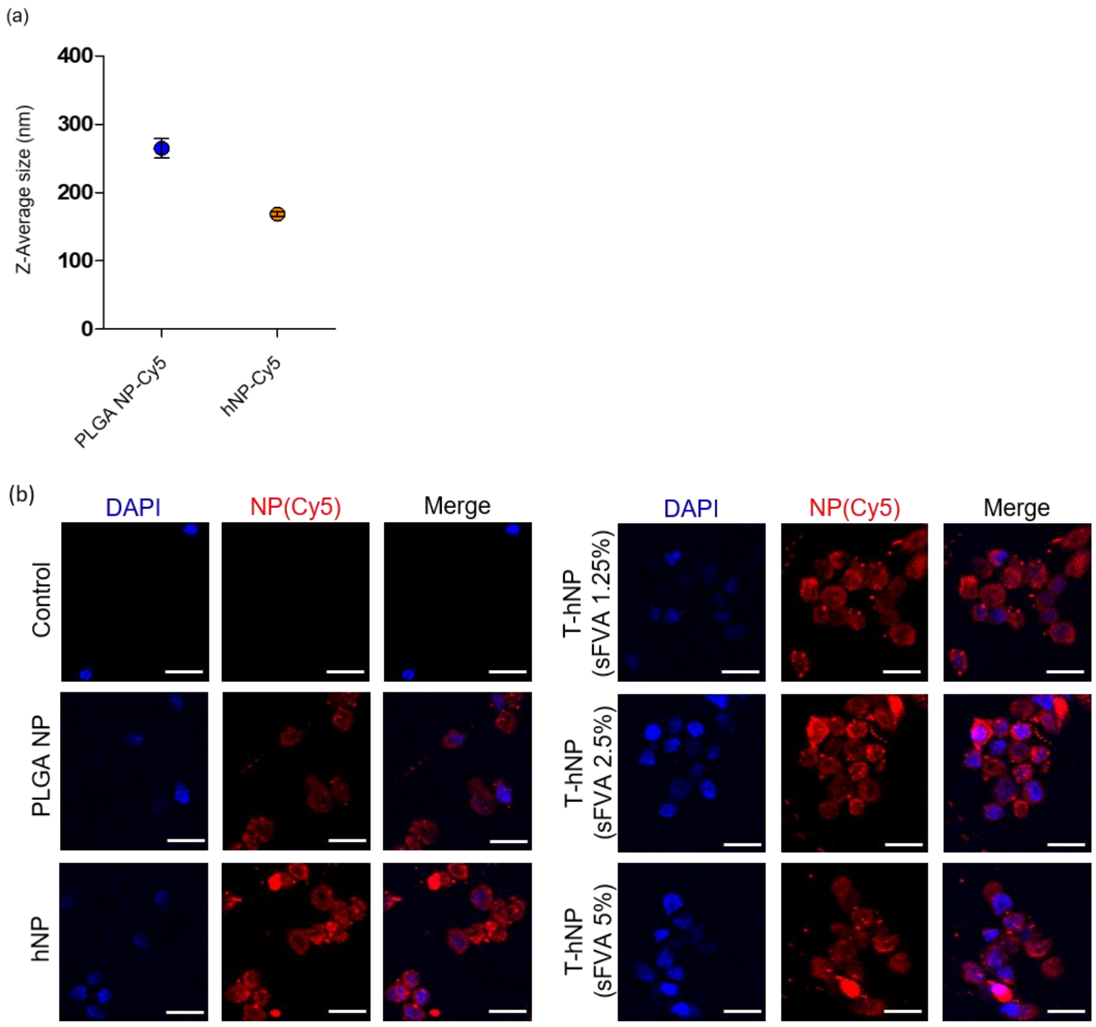

Supplementary Figure 1. Preparation of Cy5-loaded lipid-polymer hybrid nanoparticle (hNP-Cy5) & confocal microscopy image for cellular uptake study.

(a) Dynamic Light Scattering analysis of Cy5-loaded PLGA nanoparticle and -hybrid nanoparticle. (b) Confocal microscopy image for cellular uptake of hNP with/without sFVA. (particle concentration: 5µg/ml, Scale bar: 20µm

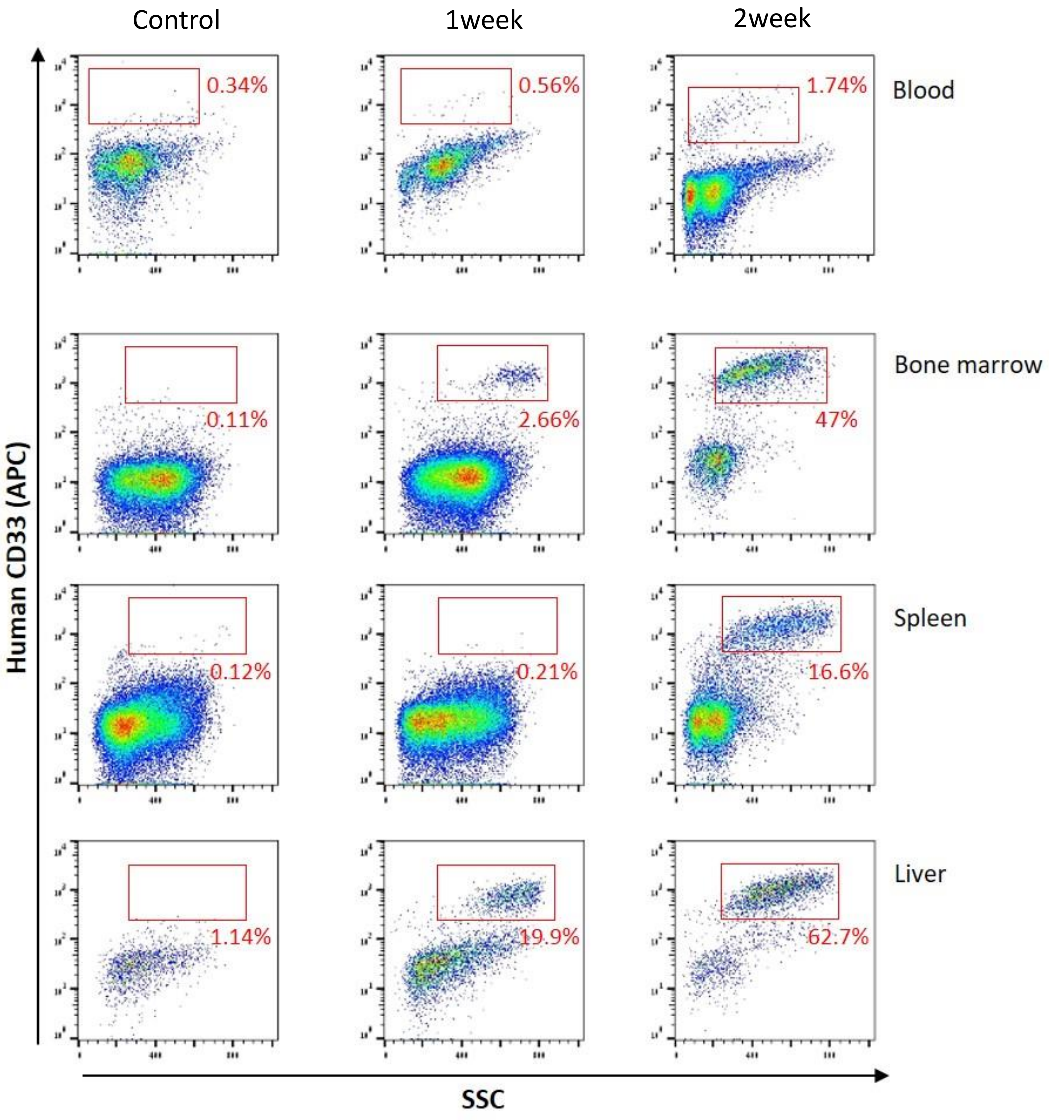

Supplementary Figure 2. Organ distribution of intravenously injected U937 cell.

(a) Flow cytometric analysis of human CD33+ U937 accumulation in leukemia niche organs. Control ;Left row, 1week after cell infusion; middle row, 2weeks after cell infusion; right row.

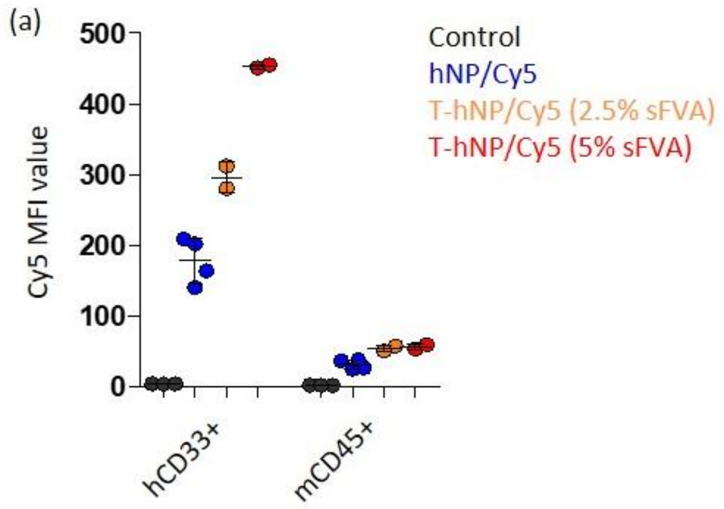

Supplementary Figure 3. Bone marrow U937-targeted delivery of hNP & T-hNP.

(a) Bone marrow cells were harvested from tibia and femur for cellular uptake analysis. Human CD33+ U937 and mouse CD45+ immune cells were analyzed for hNP and T-hNP internalization.

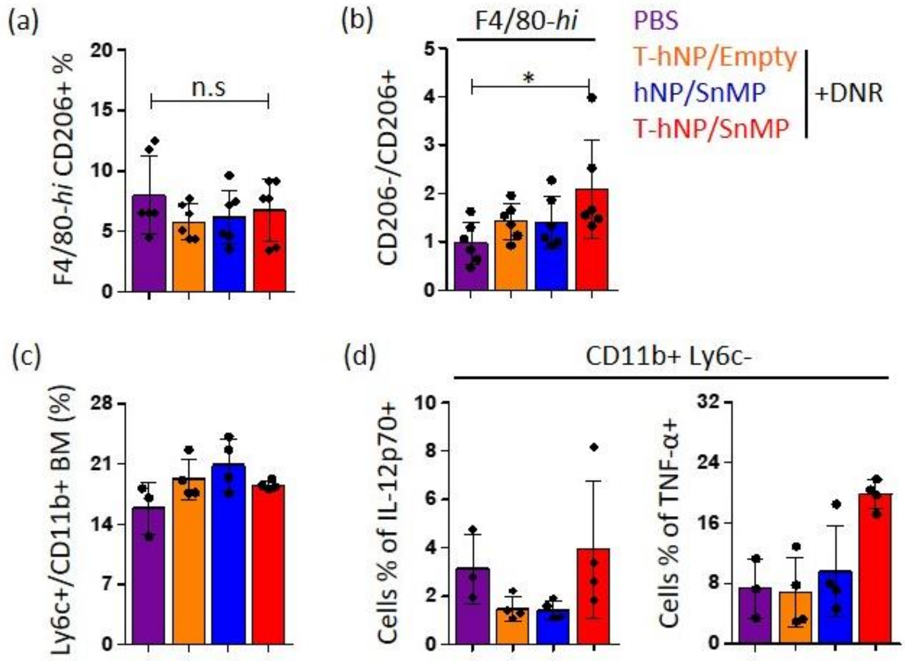

Supplementary Figure 4. Ly6c+ monocytes & Ly6c- myeloid cell analysis in bone marrow.

(a) Cells % of F4/80-hi, CD206+ M2-like macrophage in total bone marrow myeloid cells. (b) The ratio of CD206- M1-like macrophage to CD206+ M2-like macrophage in bone marrow myeloid cells. (c) Ly6c+ cells % to total CD11b+ bone marrow cells. (d) intracellular cytokine expressions in CD11b+ Ly6c- bone marrow myeloid cells.

Table 1. List of antibodies

Target	Clone name	Conjugate
Human CD33	WM53	PE, APC
Human CCR2	48607	PE, Biotin
Human CD64	M22, 10.1	FITC, PerCP-Cy5.5
Mouse CD11b	M1/70	FITC, PE
Mouse CD45	30-F11	FITC, PerCP-Cy5.5
Mouse F4/80	BM8	PerCP-Cy5.5
Mouse CD206	MR5D3	APC
Mouse Ly6c	AL-21	PE
Mouse Gr1	RB6-8C5	FITC
Mouse TNF-α	MP6-XT22	APC
Mouse IL12p70	C15.6	APC
Rat IgG1 Isotype	R3-34	APC
Mouse CD16/32	2.4G2	-
